# Subglottic web: A rare cause of respiratory distress in neonate

**DOI:** 10.4103/0971-9261.57702

**Published:** 2009

**Authors:** Sangram Singh, Mayank Pancholi, Anupama Negi, Vigya Chaurishi, Tanmay Vyas

**Affiliations:** Department of Paediatric Surgery, Sri Aurobindo Institute of Medical Sciences, Indore, MP, India

**Keywords:** Laryngeal webs, tracheostomy, subglottic web

## Abstract

A full term male neonate presented with stridor and respiratory distress at birth. Direct laryngoscopy after tracheostomy and patient stabilization revealed a subglottic web with a very small hole at 6 o'clock position. The patient was managed by cauterization of web using Bugbee electrode with resultant normal lumen post operatively.

## INTRODUCTION

Respiratory distress is common in neonates; it could be due to pulmonary, extra pulmonary and structural factors. Subglottic webs are rare and a timely tracheostomy may be life saving. Stabilization of the infant and early recognition of the etiology helps minimize complications and ensures appropriate definitive therapy.

## CASE REPORT

A full term, 2.6 kg, boy delivered by normal vaginal delivery presented with stridor and respiratory distress soon after birth; he was unable to maintain saturation even with high flow oxygen through mask. Laryngoscopy showed normal vocal cords and laryngeal opening. Bedside chest and neck roentgenograms did not suggest any abnormality. An endotracheal intubation could not be done even with the smallest endotracheal tube available. An emergency tracheostomy was performed which relieved respiratory distress. A week after tracheostomy, direct laryngoscopy revealed a subglottic web with a small hole at 6 o'clock position. The web was cauterized using Bugbee electrode. A size 3 endotracheal tube was kept as stent and removed on post operative day 3; the patient was discharged from hospital on day 7. The infant is under regular follow-up and is doing well.

## DISCUSSION

Congenital laryngeal webs are formed during embryogenesis of the laryngotracheal groove. Actively proliferating epithelium temporarily obliterates the developing laryngeal opening. The lumen is normally reestablished as the vocal cords appear separately on each side. Laryngeal web, subglottic stenosis and congenital laryngeal atresia result from different degrees of failure of the epithelium's resorption during the seventh and eighth weeks of intrauterine development. Most laryngeal webs occur at the glottic level and affect the vocal cords. More than 90% of laryngeal webs are located anteriorly and extend towards the arytenoids. The webs vary in thickness from a thin structure to one that is thicker and more difficult to eradicate. Congenital laryngeal webs are uncommon, constituting five per cent of all congenital laryngeal lesions. Many anterior webs are associated with deletions of chromosomes 22q11. Symptoms of laryngeal webs are present at birth in 75% of patients, and within one year in all patients. Major clinical features are abnormal cry or voice, respiratory distress, and croup. Cohen[1] divided laryngeal webs (glottic webs) into four types based on their appearance and an estimation of the degree of airway obstruction. Type 1 glottic webs are uniform in thickness with no subglottic extension, compromises < 35% of the airway, there is usually no airway obstruction. Type 2 glottic webs are slightly thicker, with a significantly thicker anterior component. Subglottic involvement is minimal. The web restricts the airway by 35 to 50% and usually causes little airway distress, unless the patient has an acute infection or is traumatized during intubation. Type 3 glottic web is a thick web; the anterior portion of the web is solid, extends into the sub glottis and the true vocal cords are not well delineated. The web restricts the airway by 50 to 75%, and obstruction is moderately severe. Type 4 glottic web is uniformly thick extending into the subglottic area with resulting subglottic stenosis, occluding 75 to 90% of the airway. Respiratory obstruction is severe, and the patient is almost always aphonic. Treatment of laryngeal webs depends on the thickness of the web. Approximately 60% of patients require surgical intervention. The type of lesion dictates the surgical approach. In general, the thinner webs are easier to treat and the result is better. The more severe webs are resistant to surgical management. Thin glottic webs alone respond well to simple incision or rupture. Some of the endoscopic treatment methods used are endoscopic dilatation, intralesional steroid injection[2] or carbon dioxide laser excision.[3,4] Schweinfurth described a single-stage, stentless endoscopic repair of anterior glottic webs.[5] Hsueh described intralaryngeal approach to laryngeal web using lateralization with silastic sheet.[6] Bugbee electrode is a very useful and handy instrument to cauterize the web with satisfactory outcome if the web is thin.
